# Evaluation of the Distortion Rate of Panoramic and Periapical Radiographs in Erupted Third Molar Inclination

**Published:** 2011-03-30

**Authors:** F. Ezoddini Ardakani, M. Zangouie Booshehri, B. Behniafar

**Affiliations:** 1 Associate Professor, Department of Oral and Maxillofacial Radiology, Faculty of Dentistry, Shahid Sadoughi University of Medical Sciences, Yazd, Iran; 2 Assistant Professor, Department of Oral and Maxillofacial Radiology, Faculty of Dentistry, Shahid Sadoughi University of Medical Sciences, Yazd, Iran; 3 Dentist, Faculty of Dentistry, Shahid Sadoughi University of Medical Sciences, Yazd, Iran

**Keywords:** Panoramic, Periapical, Radiographic Distortion, Third Molar, Tooth Eruption

## Abstract

**Background/Objective:**

Panoramic and periapical radiographs are normally used in impacted third molar teeth surgeries. The aim of the present study was to evaluate and compare the distortion of the erupted third molar teeth on panoramic and periapical radiographs.

**Patients and Methods:**

A total of 44 radiographs were obtained of 22 patients (age range, 18-24 years) referred to the faculty of dentistry for orthodontic treatment. A plaster cast was prepared and panoramic radiography was taken for all patients to plan the orthodontic treatment and periapical radiography was taken for investigation of tooth structure details. Therefore, a total of 66 views and samples were studied by two methods: 1) Measuring the angle between the longitudinal plane of the third molar and occlusal plane. 2) Measuring the angle between the longitudinal plane of second and third molar. Finally, 132 records were evaluated by one individual.

**Results:**

There was no significant statistical difference between the mean position of the third molar on panoramic, periapical radiographs and the casts. However, measurements of the third molars on periapical radiographs were slightly closer to the measurements of the casts compared to the panoramic radiographs.

**Conclusion:**

Distortion does not have a specific effect on the diagnosis of the position of the third erupted molars by periapical or panoramic radiographs, though various studies have shown that these radiographs have an amount of distortion and periapical radiographical distortion is less than that in panoramic radiography.

## Introduction

Conventional radiographs such as panoramic and periapical radiographs used for the management of tooth surgery and orthodontic treatment yield limited information because of the two-dimensional nature of images, produced magnification errors and dimensional distortion.[1][2][3]

Periapical radiographs are still most commonly exposed during dentistry and orthodontic procedures, providing useful information for the presence and location of periradicular lesions and the proximity of adjacent anatomical structures. Despite their widespread use, periapical images yield limited information.[2]

CT and Cone Beam CT techniques permit imaging of anatomic osseous structure in three planes, true to scale and without overlay or distortion showing the best imaging quality.[4][5][6]

Important features of the tooth and its surrounding tissues are visualized in the mesio-distal plane only. Similar features presenting in the bucco-lingual plane may not be fully appreciated.[7]

Panoramic and periapical radiographs are usually used during surgery of impacted third molar teeth to study the condition of the teeth with regard to the angle and direction of the third molar in relation to the occlusal plane or to the second molar direction. Although panoramic radiography is used in the location of the third molars, extensive diseases and developmental anomalies, it cannot present the details like periapical radiographs.[8] The dentist should be aware of the limitations of both radiographs.[9] Even with the best intentions and refined techniques, images acquired using conventional radiographs reveal information in two-dimensions only. Valuable information in the third dimension (depth) is limited.[2]

Inclination is defined as a slope, diversion, bending and deviation angle of a linear line. In dentistry, inclination of a tooth in the perpendicular plane may be mesial, distal, lingual, buccal or labial.[9]

The aim of the present study was to determine the rate of erupted third molar teeth distortion and inclination in panoramic and periapical radiographs.

## Patients and Methods 

A total of 44 radiographs (panoramic and periapical) were obtained of 22 patients, 10 men and 12 women (age range, 18-24 years) referred to the dental faculty for orthodontic treatment. The participants were recruited sequentially from February 2008 to June 2008. In general, plaster cast is prepared and panoramic radiography is taken for all patients to plan the orthodontic treatment. Then if the orthodontist recommends that surgical extraction of the third molar is necessary, periapical radiography will be taken for investigation of tooth structure details. All radiographs were taken by one operator in one radiological center. The inclusion criteria were the orthodontic treatment, presence of 2^nd^ and 3^rd^ molars and surgical extraction of the third molars with no evidence of developmental abnormalities. The exclusion criteria were existence of diseases which contra-indicate tooth surgery.

Panoramic dental images were acquired with a Planmeca 2002 EC proline multitomographic X-ray unit (Planmeca Co., Helsinki, Finland). They were obtained with a constant 12 mA, 80 kVP and 18 s exposure through 2.5 mm Al filtration. Regular Kodak Lanex (Eastman Kodak Co, Rochester, NY) intensifying screens (15×30 cm cassette) and Kodak T Mat G films (Eastman Kodak Co, Rochester, NY) were used in this study. Films were developed in an automatic film processor (Velopex, Extra-X, Medivance Instruments Ltd, London, UK) with standard solutions. The total time of processing was 4 minutes at 27oC working temperature. All panoramic radiographs were assessed by a single observer who was an oral and maxillofacial radiologist with more than 10 years of experience.

Periapical intraoral radiology were taken with planmeca proline (Finland-Helsinki) with maximum 70kv, 8mAmp and 0.16-0.25 seconds, Kodak periapical film number 2. All the radiographs were taken by one technician.

A 500 gm alginate packet named Cavex CA37 (normal set/dust free) from Cavex Holland BV, Haarlem, the Netherlands was used. 18 gm of this corresponding to two spoonfuls for the medium trays.

Zhermack plaster made in Rovigo, Italy; a specific amount of water and plaster was mixed in a plastic vessel to form a smooth and creamy consistency. Approximately 45 minutes was required for the setting of the plaster.

The panoramic and periapical radiographs were evaluated by an oral and maxillofacial radiologist using a view box in order to determine the required angles with two methods.

In the present study, 22 patients with indications for extraction of the third erupted molars were included. Periapical and panoramic radiography and cast were taken from each patient. Therefore, a total of 66 views and samples were studied by two methods:

1. Measuring the angle between the horizontal plane of the third molar and the occlusal plane (periapical 1, panoramic 1 and cast 1).

2. Measuring the angle between the horizontal plane of the second and third molar (periapical 2, panoramic 2 and cast 2).

Finally, 132 records were evaluated in two groups by one individual.

**Fig. 1:**
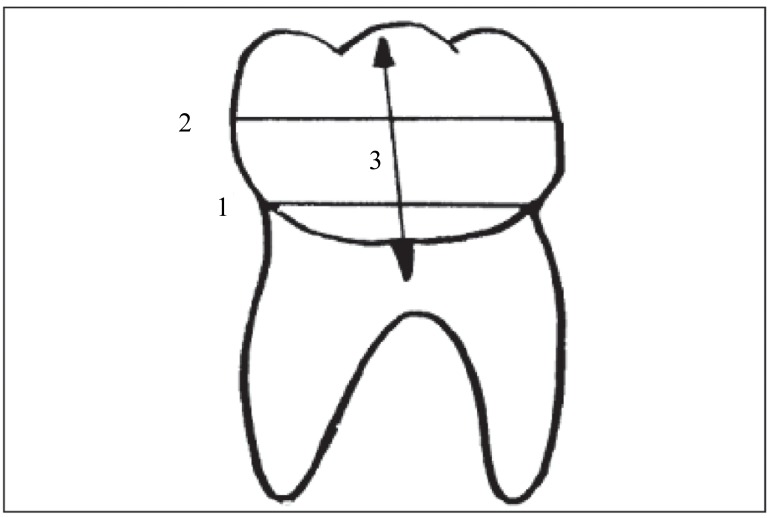
Lines 1, 2 and 3 show the horizontal lines of the third molar.

**Fig. 2:**
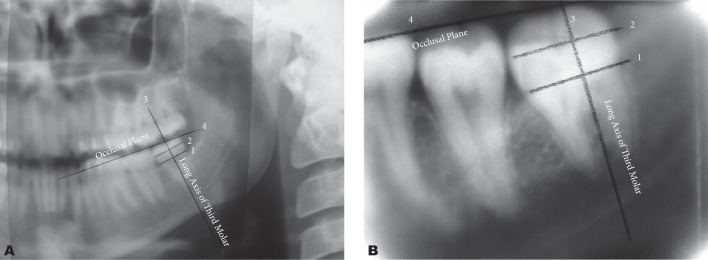
A & B. Drawing of occlusal plane and horizontal line of the third molar on panoramic and periapical radiography.

In the first step, which was to measure the angle between the longitudinal plane of the third molar and the occlusal plane, a ruler and protractor were used to plot four lines, which were namely the mesial cemento enamel junction to the distal of the third molar; the junction of the height of contour in the mesial and distal of the third molar; a vertical line on these two lines on the third molar (as the longitudinal plane of the tooth); plotting of the occlusal plane, a line passing through the biggest second and third molar cusps (Fig. 1). Then the angle plotted between line 3 and 4 on the radiographs was calculated and recorded (Fig. 2)

**Fig. 3:**
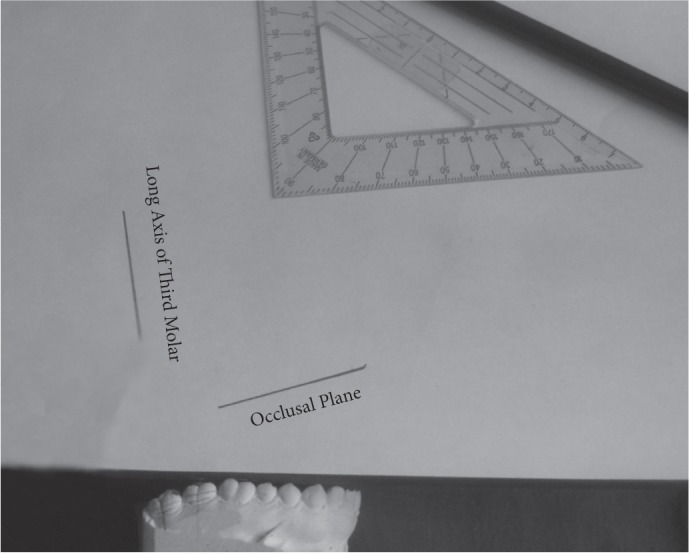
Drawing of occlusal plane and horizontal line of the third molar on cast.

In order to measure these angles on the cast, the four mentioned lines were plotted by a graphite pencil on the cast. The cast was made stable by placing it on a plain surface at a height of approximately 7 cm from the floor such that the tips of the crowns of the anterior teeth and the premolars rested on this surface. Then a transparent ruler was used and the lines were plotted on an A4 paper. The angle between the longitudinal plane of the third molar and the occlusal plane was measured and recorded (Fig. 3).

**Fig. 4:**
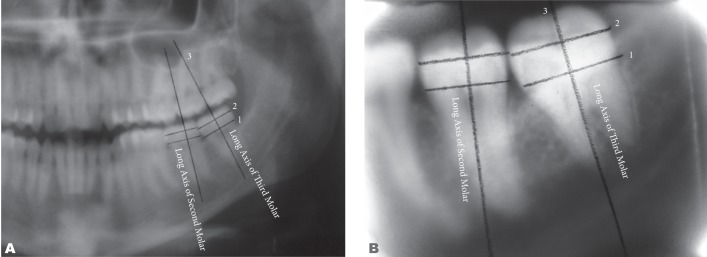
A & B. Drawing of the horizontal line of second and third molars on panoramic and periapical radiographs.

A second method also involved the use of a ruler and protractor in order to measure the angle between the longitudinal plane of the third molar and the longitudinal plane of the second molar. Three lines like the previous method were plotted and the angle between longitudinal planes of the two teeth were calculated in both radiographs (Fig. 4). The first line was the cemento-enamel junction from the mesial to the distal of the third and second molars separately. The second line was the junction of height of contour from the mesial to the distal of the third and second molars separately. A vertical line on these two lines on the third and second molars (as the longitudinal plane of the tooth).

**Fig. 5:**
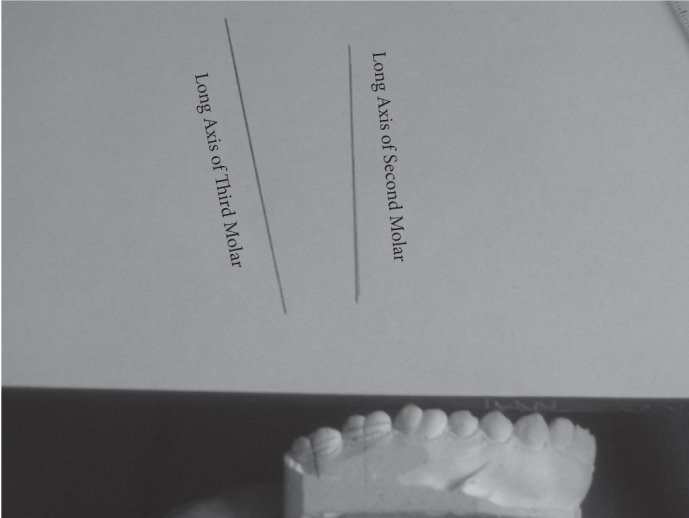
Drawing of the horizontal line of second and third molars on cast.

In order to measure these angles on the cast, the three mentioned lines were plotted by a graphite pencil. The cast was stabilized like the first method. Then using a transparent ruler, the lines were plotted on an A4 paper and the angle between the longitudinal planes of the second and third molars was measured and recorded (Fig. 5).

The gathered data included two types of angles; 1 and 2, which were derived from the panoramic and periapical radiographs and the casts as the control. Each view had 22 inputs for each angle. In order to determine the maximum distortion from the control group, each view was calculated on the basis of the type of angle and compared with the control group. The distortions were then compared. Intra-observer reliability was 0.7486 in the first method (unbiased estimate of reliability) and 0.5624 in the second method and total was 0.8219. This difference was not statistically significant.

SPSS 17 for Windows (SPSS Inc., Chicago, Illinois) used to evaluate the gathered data. For statistical analysis, paired t test, Pearson correlation coefficient and kappa statistics were used.

## Results

Totally, 22 third molar samples were evaluated on panoramic and periapical radiographs and casts by the following two methods; the angle between the third molar longitudinal plane and the occlusal plane. The angle between the longitudinal plane of the third molar and the longitudinal plane of the second molar. The distribution of the samples is shown in Table 1. The mean and standard deviation of each view is presented in Table 2. Then in order to compare the distortions of each view of the periapical and panoramic radiographs with the actual ones (cast), because the data of each view were dependent and followed the normal distribution pattern, paired t test was used for comparison and the mean difference (MD) of the data of both views in both methods were compared (Table 2).

**Table 1:** Distribution of the Various Teeth Under Study

**Type of Teeth**	**Number**	**Percentage**
Right Upper Third Molar	6	27.3
Right Lower Third Molar	5	22.7
Left Upper Third Molar	5	22.7
Left Lower Third Molar	6	27.3
Total	22	100

**Table 2:** The Angle of Panoramic and Periapical Radiographs and Comparison Between Radiographs and Casts in Two Different Methods

	**Third Molar & Occlusal**		**Third Molar & Second Molar**	
	**Mean**	**SD**	**Mean**	**SD**
Cast	87.59	9.64	9.59	4.1
Panoramic Radiography	89.45	8.55	10.05	4.96
Difference: Cast and Panoramic	1.86	5.79	0.46	5.24
P Value	0.149	-	0.688	-
Cast	87.59	9.64	9.59	4.1
Periapical Radiography	86.86	9.93	9.86	3.96
Difference: Cast and Periapical	0.73	2.91	0.27	1.56
P Value	0.255	-	0.52	-

According to Table 2, in the first method, the least MD was between periapical and cast. This value is only 0.73 degree (0.8%) less than the standard value. The 95% confidence interval of this value was from -1.94 to +0.48 degree. Paired t test was applied to measure the difference between the measurements from periapical radiographs and cast. This difference was not statistically significant (p value=0.255).

The difference between panoramic radiography and cast was 1.86 degrees, which was not statistically significant (p value=0.146). Since this difference was higher than the periapical and standard value; therefore, periapical radiography was better.

None of the radiographs had a meaningful difference with the cast (actual measurement). Similarly, there was no significant difference between the means of both periapical and panoramic radiographs. But it may be said that periapical radiographs were slightly closer to the actual values (cast).

In addition, in the second method there was a minimal difference between the mean values of periapical 2 and panoramic 2 radiographs, while there was maximum difference between panoramic 2 and cast 2 measurements. Comparing each of the radiographs with the casts, the difference between periapical 2 and cast 2 measurements was less than the difference between panoramic 2 and cast 2 measurements. This difference was not statistically significant. In other words, there was no meaningful difference between the mean of the two radiographs. It may only be concluded that periapical 2 radiographic measurements were slightly closer to the cast measurements in comparison to panoramic 2 measurements.

This value is only 0.27 degree less than the standard value. Paired t test was applied to measure the difference between the measurements from periapical radiographs and cast. This difference was not statistically significant (p value=0.52).

The coefficient correlation between the radiographs in method 1 is shown in Table 3.

**Table 3:** Correlation Coefficient Between Various Views in the First Method of Measurement

		**Panoramic 1**	**Periapical 1**
Cast 1	Pearson Correlation	0.804	0.956
	P Value	0.000	0.0001
Panoramic 1	Pearson Correlation	1	0.771
	P Value	-	0.0001

The coefficient correlation value between the measurement index of the periapical view with the cast view was (r=0.956) which is significant (p value<0.0001); therefore, the cast view has the closest measurement to the real value. The correlation coefficient between the radiographs in method 2 is shown in Table 4.

**Table 4:** Correlation Coefficient Between Various Views in the Second Method of Measurement

		**Panoramic 2**	**Periapical 2**
Cast 2	Pearson Correlation	0.343	0.883
	P Value	0.119	0.000
Panoramic 2	Pearson Correlation	1	0.257
	P Value	-	0.249

## Discussion

In dentistry, panoramic and periapical radiographs are among the main methods for obtaining data about teeth and surrounding tissues. As it is possible that panoramic radiography may be used in order to get the largest possible view of the area around the teeth under consideration, it must be noted that according to the literature, use of periapical radiography may give details of the teeth and the surrounding structures and therefore its use as a supplement to panoramic radiography may help the diagnosis.[9]

As it is possible that radiographic distortion can affect the decision of the dentist or maxillofacial surgeon during surgery of the impacted third molar teeth or even extraction of the tooth,[9] the authors studied the amount of panoramic and periapical radiographics distortion in relation to actual samples (casts) of erupted wisdom teeth and compared the two distortions.

According to the results of the first method, there was a minimal difference between the mean of cast 1 and periapical 1 radiographs, maximum difference between periapical 1 and panoramic 1 radiographs and a moderate difference between panoramic 1 and cast 1 results. But none of the above differences were statistically significant. In fact, it may be concluded that periapical 1 radiography results were slightly closer to cast 1 results.

According to the results of the second method, there was a minimal difference between the mean of periapical 2 and panoramic 2 radiographs, maximum difference between panoramic 2 and cast 2 radiographs and a moderate difference between panoramic 2 and cast 2 results. But none of the above differences were statistically significant. In fact, it may be concluded that periapical 2 radiography results were slightly closer to cast 2 results.

The correlation coefficient was significant in all of the views in the first method.

Thanyakarn et al.[10] studied the length of the first maxillary molars and mandibular premolars in panoramic radiographs of 64 extracted teeth and the actual length and radiographic length of the teeth were measured. The vertical enlargement of the mandibular premolars was less than that of the second maxillary premolars and first maxillary molars in panoramic radiography. In the present study, the measurements on panoramic and periapical radiographs were compared with actual measurements (cast) and it was concluded that even though the differences were not statistically significant, distortions of panoramic radiographs were slightly more than that of periapical radiographs.

Batenburg et al. studied the rate of distortion of panoramic radiographs on the angles, position and shape of toothless mandibles. They concluded that panoramic radiographs are not ideal for evaluation and diagnosis of toothless mandibles.[11] But in the present study, even though there were differences in the measurements on panoramic and periapical radiographs and the actual measurements, they were not statistically significant and worth consideration. This difference in conclusion of the two studies could be due to the difference in the method of study and number of cases as in the Batenburg study, only five dry toothless mandibles were studied, while in the present study, 22 erupted wisdom teeth in live subjects were studied.

Presson et al. compared panoramic and intra oral radiographs in order to determine the level of the alveolar bone. They concluded that the results of panoramic and intra oral radiographs are close to each other, but it is better to use panoramic radiographs and ultimately replace them with full mouth periapical films.[12] In the present study too, there was no meaningful difference between panoramic and periapical (intra oral) radiographs, but the results of periapical radiography were slightly closer to the actual measurements, demonstrating that the enlargement and distortion levels were less in them.

Sant’ Ana et al.[3] evaluated the distortions in panoramic radiographs of third mandibular molars. Ultimately, a difference of approximately -5.37 degrees was observed between the position of the third molars on the panoramic radiography and cast samples. There is a distortion in the position of the third molar in panoramic radiography that can affect the surgeon’s decision.[9] This study was much similar to the present study, but the difference in distortion in the present study was not statistically significant. The difference in results could be due to the difference in the type of machines or the type of cases studied which were erupted molars in the present study and impacted teeth in that study.

Laster et al. measured skulls with ideal and bad positions in order to evaluate the errors in measurement occurring in panoramic radiographs. They concluded that panoramic radiographs should be used carefully in important measurements or relative comparisons. It is important to note this point that the enlargements of panoramic radiography are different in different machines.[13]

Therefore, in this study, evaluation and comparison of the distortion of each of the techniques was done and it was concluded that there is no significant difference between these pictures and the actual measurements.

Volchansky et al. compared the vertical and horizontal measurements of the posterior teeth in panoramic and periapical radiographs. They compared the standard panoramic and periapical radiographs of 16 human skulls along with ball bearings placed on the first upper molars in order to measure the vertical and horizontal magnification. In 14 cases, there was no difference between the two radiographs while in four areas, the vertical measurement was more in the panoramic film (0.8-1.37) and in one area, the horizontal measurements were more in the periapical films (0.88).[14]

Durta et al. evaluated the mental index of panoramic radiography. They took panoramic radiographs from several dried mandibles. The samples were cut vertically in the mental region and the thickness of the cortical bone of mandible was measured using vernier caliper. The measurements were compared with the radiographic panoramic measurements and the results indicated that panoramic radiography is accurate.[15]

In conclusion, considering the comparison of the two techniques, it seems that in cases of evaluation before surgery where high clarity and low distortion is required, it is better to use periapical radiographs along with panoramic radiographs.
